# Dietary Intake and Food Sources of Niacin, Riboflavin, Thiamin and Vitamin B_6_ in a Representative Sample of the Spanish Population. The ANIBES Study †

**DOI:** 10.3390/nu10070846

**Published:** 2018-06-29

**Authors:** Juan Mielgo-Ayuso, Raquel Aparicio-Ugarriza, Josune Olza, Javier Aranceta-Bartrina, Ángel Gil, Rosa M. Ortega, Lluis Serra-Majem, Gregorio Varela-Moreiras, Marcela González-Gross

**Affiliations:** 1 ImFINE Research Group, Department of Health and Human Performance, Technical University of Madrid, 28040 Madrid, Spain; juankaya@msn.com (J.M.-A.); apariciougarriza.raquel@gmail.com (R.A.-U.); 2 Department of Biochemistry, Molecular Biology and physiology of University of Valladolid, 42003 Soria, Spain; 3 Department of Biochemistry and Molecular Biology II, and Institute of Nutrition and Food Sciences, University of Granada, 18100 Granada, Spain; jolza@ugr.es (J.O.); agil@ugr.es (Á.G.); 4 CIBEROBN (Physiopathology of Obesity and Nutrition CB12/03/30038), Carlos III Institute (ISCIII), 28029 Madrid, Spain; 5 Department of Preventive Medicine and Public Health, University of Navarra, C/Irunlarrea 1, 3100 Pamplona, Spain; jaranceta@unav.es; 6 Department of Nutrition and Food Science, Faculty of Pharmacy, Complutense University of Madrid, Plaza Ramón y Cajal s/n, 28040 Madrid, Spain; rortega@ucm.es; 7 Research Institute of Biomedical and Health Sciences, University of Las Palmas de Gran Canaria, C/Doctor Pasteur s/n Trasera del Hospital, 35016 Las Palmas, Gran Canaria, Spain; lluis.serra@ulpgc.es; 8 Department of Pharmaceutical and Health Sciences, Faculty of Pharmacy, CEU San Pablo University, 28668 Madrid, Spain; gvarela@ceu.es; 9 Spanish Nutrition Foundation (FEN), 28010 Madrid, Spain

**Keywords:** ANIBES study, b-related vitamins, misreporting, food intake

## Abstract

Thiamin, riboflavin, niacin, and vitamin B_6_ are essential micronutrients that are mainly involved in energy metabolism; they may prevent the occurrence of developmental abnormalities and chronic degenerative and neoplastic diseases. The aim was to analyze dietary intake and food sources of those four nutrients in subjects (*n* = 2009) aged 9–75 years old from the Spanish ANIBES (Anthropometric data, macronutrients and micronutrients intake, practice of physical activity, socioeconomic data and lifestyles in Spain) study. Dietary data were collected by means of a validated, photo-based three-day dietary food record. Underreporting was analysed according to the European Food and Safety Authority (EFSA, Parma, Italy) protocol. Mean (max–min) reported intake for the whole population of thiamin was 1.17 ± 0.02 mg/day, (0.30–3.44 mg/day), riboflavin 1.44 ± 0.02 mg/day, (0.37–3.54 mg/day), niacin 29.1 ± 0.2 mg/day (6.7–109 mg/day), and vitamin B_6_ 1.54 ± 0.01 mg/day (0.28–9.30 mg/day). The main sources of intake for thiamin, niacin, and vitamin B_6_ were meat and meat products, and for riboflavin were milk and dairy products. An elevated percentage of the Spanish ANIBES population meets the EFSA recommended intakes for thiamin (71.2%), riboflavin (72.0%), niacin (99.0%), and vitamin B_6_ (77.2%).

## 1. Introduction

In recent years, due to growing evidence that some B vitamins may prevent the occurrence of developmental abnormalities and chronic degenerative and neoplastic diseases, special consideration has been given to the possible use of such indicators as criteria of adequacy [1]. Moreover, the B-complex vitamins are especially important for the energy metabolism. Concretely, thiamin, riboflavin, niacin, and vitamin B_6_ are required for decarboxylation, transamination, acylation, oxidation, and reduction of substrates that are ultimately used in energy consumption. One or more of these are also important for amino acids, fatty acids, cholesterol, steroids, and glucose synthesis [2].

In fact, thiamin plays a critical role in the energy metabolism, and, therefore, in the growth, development, and function of cells [3]. Although thiamin deficiency is not usual in developed countries, because it is contained in breads, cereals, pulses, meat, liver, fish, and some fortified foods [4], the existence of thiamin deficiency has been observed in more than half of the elderly people who require nursing care [5,6].

Riboflavin is an essential component of two coenzymes that play major roles in energy production; cellular function, growth, and development; and, metabolism of fats, drugs, and steroids [7,8]. Riboflavin deficiency is extremely rare in developed countries because its sources are commonly consumed foods (milk and milk drinks, bread and bread products, mixed foods whose main ingredient is meat, ready-to-eat cereals, and mixed foods whose main ingredient is grain) [7,8]. However, adolescents are at a high risk for nutritional deficiencies because of their notoriously poor dietary habits, and the estimation of riboflavin deficiency may be an indicator of the overall nutritional status [9].

The function of niacin in the human body is the precursor of the nicotinamide nucleotide coenzymes NAD and NADP. NAD is essential for energy-producing reactions and NADP for anabolic reactions. Moreover, NAD also participates in unique non-redox adenosine diphosphate–ribose transfer reactions that are involved in protein modification, calcium mobilization, cell signaling, and DNA repair [10,11]. Although good sources of niacin are liver, meat and meat products, fish, peanuts, and whole grains [12], deficiency in niacin intake has been observed in European preschoolers [13].

Finally, vitamin B_6_ in coenzyme form performs a wide variety of functions in the body and it is extremely versatile, with involvement in more than 100 enzyme reactions, which are mostly concerned with protein metabolism [7,14]. Vitamin B_6_ is found in a wide variety of foods. The richest sources of vitamin B_6_ include fish, beef liver and other organ meats, potatoes and other starchy vegetables, and fruit (other than citrus). About 75% of vitamin B_6_ from a mixed diet is bioavailable [7,14]. Although vitamin B_6_ deficiency is uncommon, the prevalence of B_6_ deficiency in young adult non-pregnant women (19–35 years) has been described in 1.5% and that of suboptimal B_6_ status in 10.9% [15].

Studying the nutritional situation of the population as well as lifestyle habits is fundamental to design national guidelines and public policies to prevent diseases. The role of diet in the decreasing progression of chronic disease is becoming increasingly important [16]. Likewise, maintaining inadequate intake for a long time could be reflected on blood concentration and this fact could have negative effects on health [17]. European Food and Safety Authority (EFSA) has published the “Guidance on the EU Menu methodology”, which facilitates the collection of more consistent food consumption data from all European Union (EU) Member States [18] and the present study is based on these guidelines. In this line, given that the data collected in the surveys are mostly based on subjects self-reporting, the surveys frequently report data that do not represent the habitual intake of the studied population and estimated energy intakes (EIs) that are not plausible physiologically [19]. On this point, EFSA has also published a protocol to identify misreporting [20]. Although previous papers have reported intake of energy [21], macronutrients [22], and some micronutrients [23,24,25] in a Spanish representative sample, as part of the ANIBES study (Anthropometric data, macronutrients and micronutrients intake, practice of physical activity, socioeconomic data and lifestyles in Spain), there is still a lack of knowledge about the intake of some vitamins involved in the energy metabolism. Therefore, in the present paper, we analyze the reported intake of niacin, riboflavin, thiamin and vitamin B_6_ in the whole population, and in the plausible energy reporters separately (following the EFSA harmonized approach to identify misreporting), and assess the food that contains them.

## 2. Materials and Methods

ANIBES is a Spanish study that evaluates energy intake and expenditure, dietary patterns and sources, and body composition in a Spanish representative sample [26].

### 2.1. Sample and Design

ANIBES was designed as a cross-sectional study through a stratified multistage sampling. The study was conducted from mid-September 2013 to mid-November 2013 and two previous pilot studies were also performed. For more coverage and representativeness, 128 sampling points across Spain were considered to guarantee better coverage and representativeness (with 90 interviewers allocated in 11 areas and 12 coordinators, all being previously trained by the Spanish Nutrition Foundation (FEN)). No previous pre-recruitment was considered, which minimized the risk of bias in responses. ANIBES Study´s sample was obtained based on 2012 census data published by the INE (Instituto Nacional de Estadística/Spanish Bureau of Statistics, Madrid, Spain) for gender, age, habitat size, and region [26]. For to define that the sample size was representative of all individuals living in Spain (excluding the autonomous cities of Melilla and Ceuta), the ANIBES study aims people aged 9–75 years and residing in cities of at least 2000 residents. The initial number of participants recruited for the ANIBES study was 2914. However, 2634 (90.3%) recorded at least three days of intakes and responded to the interview on the second visit. Nevertheless, once all of the monitoring and cleaning procedures were applied, the number of valid participants was 2285 (78.4% of the initial sample). Concretely, the final sample comprised 2009 people aged 9–75 years (1013 men, 50.4%; 996 women, 49.6%) (2.23% error and 95.5% confidence interval) [26]. In addition, for the youngest groups (9–12, 13–17, and 18–24 years old), and oldest (65–75 years) a boost was considered in order to have at least an *n* = 200 per age group (error ±6.9%). Therefore, the random sample plus booster was 2285 participants [26].

Subjects excluded in the study were: individuals living in an institutional setting; individuals following a therapeutic diet due to recent surgery or determined pathologies; potential participants with any transitory illness at the time fieldwork was undertaken; and, individuals employed in areas related to consumer science, marketing, or the media. Likewise, individuals under the following conditions were considered to be eligible for inclusion: those following dietary protocols, such as for the prevention of hypertension, diabetes, hypercholesterolemia, or hyperuricemia; pregnant and lactating females; individuals with diagnosed allergies and/or food intolerance; or, those with metabolic disease, for instance, hyperthyroidism, or hypothyroidism. The sample quotas according to the following variables were age groups: 9 to 12 (children), 13 to 17 (adolescents), 18 to 64 (adults), and 65 to 75 (elderly); sex (men/women); geographical distribution (North-East, Levant, South, West, North-Central, Barcelona, Madrid, Balearic, and Canary Islands); habitat size: 2000 to 30,000 inhabitants (rural population); 30,000 to 200,000 inhabitants (semi-urban population); and, over 200,000 inhabitants (city/town population). Additionally, other factors for sample adjustment were considered: rate of unemployment, percentage of foreigners (immigrant population), level of physical activity, and education/economical level [26].

The final protocol was approved by the Ethical Committee for Clinical Research of the Region of Madrid (Spain).

### 2.2. Food Record and Adequacy of Reported Intake

Participants were provided with a tablet device (Samsung Galaxy Tab 2 7.0, Samsung Electronics, Suwon, Korea). They were also trained to record all information related to food and drink, before beginning to eat and drink, again after finishing for two weekdays and one weekend day. In addition, they were asked to record a brief description of foods, recipes, brands, and other relevant information on the tablet. In the same way, participants also recorded the brands and doses of the dietary supplements. Participants who showed problems with using the tablet device were offered other options, such as the use of a digital camera, a paper record, or telephone interviews. In this sense, 79% of the sample used a tablet, 12% a digital camera, and 9% a telephone interview. Food records were coded by trained coders and supervised by dietician-nutritionists. An ad hoc central server software/database was developed for this purpose to work in parallel with the coding and verification processes [26]. Energy and nutrient reported intakes were calculated from food consumption records using VD-FEN 2.1 software (FEN, Madrid, Spain), which is a Dietary Evaluation Program from the Spanish Nutrition Foundation. The program was developed by the FEN for the ANIBES study from Spanish food composition tables [27]. Data was obtained from food manufacturers and nutritional information provided on food labels was also included. A food photographic atlas was used to assist in assigning weights to portion sizes. Reported intake data was compared with European daily recommendations of niacin [12], riboflavin [8], thiamin [4], and vitamin B_6_ [14]. The disparity between reported consumption and the level needed for adequacy was calculated when comparing with 80% of EFSA population reference intake (PRI) or adequate intake (AI), according to Moreiras et al. [27] and used in other works related to vitamins and minerals in the Spanish population [23,24,25,28].

### 2.3. Evaluation of Misreporting

In the present research, EFSA protocol was used to assess misreporting [18], which has also been detailed previously [23]. EFSA recommends that the data should be reported for the complete population as well as divided into plausible and non-plausible reporters, as we do in the current paper. The method that was proposed by EFSA compares the reported energy intake (EIrep) as compared to the supposed energy requirements. EIrep is expressed as a multiple of the mean basal metabolic rate estimated (BMRest), and it is compared to the presumed energy expenditure of the studied population. Subsequently, the ratio EIrep:BMRest is referred to as the physical activity levels (PAL); PAL is established for young people (≤17 years) and adults (≥18 years) in three levels: low (1.6 and 1.4); moderate (1.8 and 1.6); and, vigorous (2.0 and 1.8). The protocol specifies that the analyses should be performed at group and individual levels. The group level determines the overall bias to the reported EI, and the individual level shows the rate of under and over reporters. To calculate the misreporting at both levels, the lower and the upper cut-off values were specifically calculated for the population. Plausible reporters were considered those individuals with estimated values of the ratio reported EIrep: BMRest ranging between calculated lower cut-off and upper cut-off values for the studied population allocated to the different category of physical activity [20]. The BMRest was calculated using the Schoefield equations [29], and the physical activity was assessed during interviews with the international physical activity questionnaire (IPAQ) [30]. Misreporting cut-offs at the group and individual levels for the ANIBES study have been detailed previously [23,24] and are shown in Table 1. CV-WEI (coefficient of variance in energy intake within-subject) for the ANIBES population was 36.6% for children and adolescents and 41.6% for adults, respectively; and, S (factor that considers the variation in energy intake, BMR and PAL) was 27.3 and 29.6 for children and adolescents and adults, respectively.

### 2.4. Statistical Analysis

Analyses were performed using IBM SPSS version 22.0 (IBM Corp., Armonk, NY, USA). Data are expressed as mean ± standard error of the mean (SEM), median, ranges, percentiles, and percentages. Normality of the distribution was assessed using the Kolmogorov–Smirnoff normality test in a random sample (2009 participants) and random plus booster sample (2285). Depending on the normality distribution, parametric or non-parametric statistical tests were used for comparisons among groups. The random sample was used to show total sample data and to compare between sexes.

A booster sample was included to enlarge those groups less represented in the random sample to compare by sex within each age group. Comparisons between groups were performed by the Mann–Whitney *U* test or a Student’s *t*-test for independent samples to evaluate differences by sex both for each age group and the whole population.

Kruskal–Wallis analysis or analyses of variance (ANOVA) tests with Bonferroni correction for multiple comparisons were performed to calculate differences among each age group. These procedures have considered the sampling complexity during the stratification of the study design. The level of significance was set at *p* < 0.05.

## 3. Results

### 3.1. Thiamin, Riboflavin, Niacin and Vitamin B_6_ Reported Intake and Distribution in the Whole Population

Table 2 depicts the daily reported intake levels of thiamin, riboflavin, niacin, and vitamin B_6_.

### 3.2. Thiamin, Riboflavin, Niacin and Vitamin B_6_ Reported Intake in Plausible and Non-Plausible Reporters

Table 3 shows the vitamin intake data divided by plausible and non-plausible reporters. The percentages of plausible reporters by age groups were as follows: children 56%, adolescents 36%, adults 26%, and elderly 22%. According to the misreporting analysis, we observed that the reported consumption of the four nutrients was significantly higher (*p* < 0.001) in the plausible reporters than in the non-plausible reporters in the whole population, as well as divided by age group and within sexes. 

### 3.3. Disparity between Reported Intake and the Level Needed for Adequacy for Thiamin, Riboflavin, Niacin and Vitamin B_6_ in the Whole Population

Table 4 shows the percentage of the entire population and the plausible energy reporters that met 80% of the European recommended daily intakes for thiamin [4], riboflavin [8], niacin [12], and vitamin B_6_ [14]. The entire population met the daily intake recommendations for niacin and vitamin B_6_; however, 28% of the population did not meet the daily intake recommendations for thiamin and riboflavin.

### 3.4. Contribution of Food Sources to Thiamin, Riboflavin, Niacin and Vitamin B_6_ Reported Intakes

The intake data was grouped into 16 food groups, 33 subgroups, and 754 ingredients for in-depth analysis.

**Figure 1:**
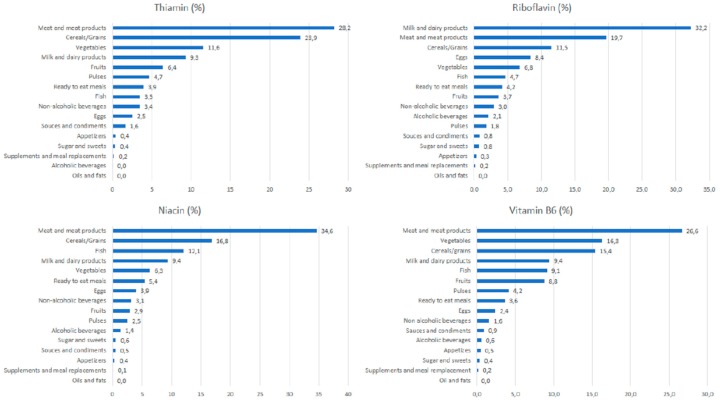
Contribution of food sources to the daily thiamin, riboflavin, niacin vitamins B6 intake in the total ANIBES population.

Figure 1 depicts the contribution (%) of food and beverage categories to the daily thiamin, riboflavin, niacin, and vitamin B_6_ reported intake for the whole population. Supplementary Figures S1–S4 show these data separately by age groups. Supplements and meal replacements contribute with 0.2% or less than the intake of the four analysed vitamins.

#### 3.4.1. Thiamin

The main sources of thiamin for the entire population were meat and meat products (28.2%; this contribution was higher in seniors, 39.1%), cereals and grains (23.9%), and vegetables (11.6%). Cereals and grains provided higher percentages to the younger groups, whereas fish did for the older groups. Milk and dairy products (9.3%), fruits (6.4%), pulses (4.7%), and ready-to-eat meals (3.9%) complete the list to reach more than the 85% of the total reported intake of thiamin. Ready-to-eat meals afforded a higher percentage in the adolescent’s group, while fish did for the older groups.

#### 3.4.2. Riboflavin

The largest source was milk and dairy products (32.2%), with a higher contribution for the children group (39.8%). Meat and meat products provided 19.7% and cereals and grains 11.5%. Eggs contributed 8.4% and vegetables 68%; this last food group afforded less in the younger groups and more in the elderly groups. Fish supplied 4.7%, and ready-to-eat meals 4.2%; the last food group contributed more to the younger groups and less to the older adults. All of these groups afforded nearly 90.0% of the riboflavin reported intake.

#### 3.4.3. Niacin

Meat and meat products were the main sources of niacin for the whole population (34.6%), contributing in higher proportions to the younger groups than to the older adults. Cereals and grains products provided 16.8% to the whole population, contributing more to the younger groups. Fish ranked third (12.1%), and next was milk and dairy products (9.4%); this last food group provided less among the younger groups and more among the older adults. Vegetables supplied 6.3%, ready-to-eat meals 5.4%, and eggs (3.9%) to the reported niacin consumption for the whole population. Except vegetables, these food groups contributed more to the younger groups and more to the older adults. All of these groups supplied nearly 90% of the total reported niacin intake.

#### 3.4.4. Vitamin B_6_

Meat and meat products was the main source of vitamin B_6_ (26.6%) for the whole population, although it contributed much less in children and senior groups than adolescents and adult groups. Vegetables provided 16.3% and cereals and grains 15.4%, with the latter food group providing a higher proportion for the older groups. Milk and dairy supplied 9.4% to the whole population, thus contributing to the largest proportion in the younger groups. These four food groups, together with fish (9.1%), contributed close to 80% of the total daily vitamin B_6_ reported intake.

## 4. Discussion

The water-soluble B vitamins thiamin, riboflavin, and niacin have important functions as part of the energy metabolism. Consequently, the reference values for the intake of these vitamins are derived in consideration of the reference values for energy intake. The present paper analyzes the daily intake of the main micronutrients that are involved in the energy metabolism. Individuals in the ANIBES who met the European recommendations for thiamin, riboflavin, niacin, and B_6_ were 71.2%, 72.0%, 99.0%, and 77.2%, respectively. Even when the plausible energy reporters were analyzed separately, these percentages remained above 90% for each vitamin.

When we compare our findings with other studies, we observe that only a few studies applied the misreporting methodology, showing extensive variability regarding underreporting. In this line, when studies used the 24-h recall method, misreporting goes from 4% to 67%, and when the food record method is used, this goes from 8% to 49% [31,32]. The typical range of misreporting observed in studies goes from 20% to 30%, thus being higher in women than in men, and more common among overweight and obese individuals [33,34]. However, our study presented a higher proportion of non-plausible reporters both in males (53.3%) and females (46.7%) [25]. In addition, while adults presented 75.9% of non-plausible reporters, in children and adolescents only 5.8% and 8.4%, respectively, was included in this group. Nonetheless, even with a lower total population that reported a plausible energy intake, we can ensure that our data are reliable and reflect the true nutrient intake situation of the Spanish population [24].

When comparing the thiamin data obtained in the present study with the data obtained in ENALIA (The National Dietary Survey on the Child and Adolescent Population in Spain) [35] for children and adolescents (two non-consecutive 24 h dietary recalls), we observed that the thiamin intake was similar for male children (1.4 mg/day) and adolescents (1.6 mg/day), as well as for women (1.3 mg/day and 1.3 mg/day, respectively). However, when ANIBES data were compared with the ENIDE (Spanish Dietary Intake Survey) (two non-consecutive 24 h dietary recalls) [36] for adults, we observed lower values in men (1.7 mg/d vs. 2.06 mg/d) and women (1.2 mg/d vs. 1.76 mg/d). In both cases, when we consider only the ANIBES plausible energy reporters, the intake of this nutrient was adequate for most of the population in these three studies when comparing with the European recommendations.

Regarding thiamin intake, other European studies indicate ranges in boys between 0.75 mg/day from the study VELS of Germany (two non-consecutive 24 h dietary recalls) [37] and 1.29 mg/kg from the NDNS of the United Kingdom (four-day dietary record) [38]. Likewise, thiamin intake ranges in girls from 0.68 mg/day to 1.29 mg/day in the same studies, respectively [37,38]. These data are lower than those obtained by our study, perhaps because in these studies children under nine years old were included. However, similar data to the ANIBES study were observed in the studies that were carried out in Germany [37,39], Finland (48 h dietary recalls) [40], France (seven days food records) [41], the United Kingdom [38], Italy (three days food record) [42], and the Netherlands (two non-consecutive 24 h dietary recalls) [43] for both male and female European adolescents. In the same way, ANIBES data from adults indicate consumption that is similar to other European studies, except for Italians [42], Dutch [37,39], and French [41], who showed lower consumption in both men and women. In relation to seniors, similarities are shown only with Finland (48 h dietary recalls) [44] and Sweden (food records-four days) [45] in both sexes, with lower values observed in the data of other countries. These lower values in the data may be produced by not eliminating those who underestimated the intake, which is a strength of the ANIBES study. Nevertheless, the different methodology that is used in the studies for recording dietary intake makes comparison between studies difficult and discrepancies, but even similarities, can be casual. Therefore, there is a need for a methodological consensus in nutritional surveys to make data comparisons between studies reliable.

Unlike the ENIDE study [36] and European studies [4] where the main source of thiamin is cereals/grains followed by meat and meat products, in the ANIBES study, the main source of thiamin is meat and meat products, followed by cereals/grains, vegetables, and milk and dairy products. This distribution is found more in the old life stages, while in children and adolescents other products appear, such as fish and ready-to-eat meals as a source of thiamin. In recent years, these changes in the Spanish population’s thiamin sources indicate that social and economic changes have led to substantial modifications in food patterns in the last decades, moving the Spanish diet away from the traditional Mediterranean Diet pattern [46].

Riboflavin intake that was obtained in this study for children and adolescents’ plausible reporters was similar to the data obtained in ENALIA [35], both in males (children: 1.8 mg/day; adolescents: 1.9 mg/day) and females (children: 1.7 mg/day; adolescents: 1.5 mg/day). In adults, data from ANIBES showed similar values to the ENIDE study [36] when we study the whole population (men: 1.5 mg/day and women: 1.4 mg/day). However, when we use only plausible reporter data, our results are higher (men: 2.0 mg/day and women: 1.6 mg/day). The intake of riboflavin is adequate for 91.3% of the total when comparing with the European recommendations, being higher in the younger group. 

Regarding the riboflavin intake in other European studies, the range in boys was between 1.1 mg/day from the study VELS of Germany [37] and 1.8 mg/kg from Finland [44]. Likewise, the riboflavin intake range in girls is from 1.0 mg/day to 1.6 mg/kg in the aforementioned countries, respectively [37,44]. Our data are in line with those observed in Finland, being higher than in other European countries [37,38,39,40,41,42,43]. In contrast, plausible female adolescent reporters from the ANIBES study showed similar data to other European female adolescents from Germany [37,39], Finland [40], France [41], the United Kingdom [38], Italy [42], and the Netherlands [43]. However, plausible male adolescent reporters from the ANIBES study presented similar data to those from Finland [40], France [41], and Italy [42], and higher than those from Germany [39], the United Kingdom [38], and the Netherlands [43].

Milk and dairy products make the greatest contribution to riboflavin intake in Western diets [47]. The same pattern was observed for both the ANIBES study and European studies [8]. After milk, meat and meat products, and cereals/grains are other riboflavin sources both in ANIBES and other European countries, such as Germany [37,39], Finland [40], France [41], the United Kingdom [38], Italy [42], and the Netherlands [43].

Niacin intake found in the ANIBES study for children and adolescents plausible reporters was similar to data found in the ENALIA study [35], both in males (children: 32 mg/day; adolescent: 41 mg/day) and females (children: 30 mg/day; adolescent: 32 mg/day). In adults, data from plausible reporters from the ANIBES study showed similar values to the ENIDE study [36] in males (41 mg/day). However, in female adults, the ANIBES study presented lower niacin intake (ANIBES: 32 mg/day and ENALIA: 35–36 mg/day) [35]. Despite these differences, niacin intake is adequate for 100% of the whole population when compared to the European recommendations [12].

Niacin intake in other European boys was similar in Italians (31.5 mg/day) [42] and in French (30.4 mg/day) [41]. Likewise, Italian girls ingested similar levels of niacin to ANIBES boys [42]. Curiously, other European children had low niacin intake [12]. On the other hand, Italian and French male and female adolescents had higher niacin intake than ANIBES adolescents. However, Dutch adolescents had lower intakes than ANIBES adolescents [43]. Italian and Finnish male adults and Dutch and Finnish [40] female adults consumed similar levels of niacin to the ANIBES adults. The rest of European adults, except for UK female adults (29.5 mg/day) [38], showed higher values of niacin intake, in some cases, values that were greater than 50 mg/day (Ireland: [48] and Sweden [45]). Finally, the elderly from Finland, Dutch (females) and the UK showed similar niacin intake to the ANIBES elderly. Vitamin B_6_ intake from the ANIBES study for Spanish children and adolescent plausible reporters was lower than from the ENALIA study [35], both in males (children: 2.0 mg/day; adolescents: 2.5 mg/day) and females (children: 1.9 mg/day; adolescents: 1.8 mg/day). Regarding Spanish adults, data from plausible reporters from the ANIBES study showed similar vitamin B_6_ intake to the ENIDE study in females (1.7 mg/day) [36]. However, male adults from the ANIBES study presented higher vitamin B_6_ intake than the ENALIA study (ANIBES: 2.2 mg/day and ENALIA: 1.98 mg/day) [35]. Despite these differences, the Spanish population from the ANIBES study met 93.4% of the European recommendations for vitamin B_6_ intake [14].

Regarding vitamin B_6_ intake, it was similar in other European boys: Finland (1.55 mg/day) [40], Germany (1.54 mg/day) [39], and in the UK (1.61 mg/d) [38]. Likewise, UK girls showed similar vitamin B_6_ intake to ANIBES girls (1.51 mg/day) [38]. However, other European children presented low vitamin B_6_ intake (boys: 1.10–1.45 mg/day and girls: 0.99–1.47 mg/day) [14]. On the other hand, French [41], German [39], Italian [42] male and female adolescents, and Dutch female [43] had similar vitamin B_6_ intake to ANIBES adolescents. However, other European countries presented higher values of vitamin B_6_ for adolescents [14]. With regard to adults, Dutch male and female adults [43] and male Finland adults [40] presented similar vitamin B_6_ intake to Spanish adults from the ANIBES study. However, male adults from Sweden (2.54 mg/day) [45], Ireland (3.09 mg/day) [48], and the UK (2.50 mg/day) [38] and female adults from Ireland (2.11 mg/day) [48] presented higher intake values of vitamin B_6_. Finally, elderly males from Sweden [45] showed similar vitamin B_6_ intake data to the elderly from the ANIBES study. On the other hand, elderly males from Ireland (2.64 mg/day) and the UK (2.54 mg/day) [38], and elderly females from Sweden (2.11 mg/day) [45] and Ireland (2.16 (mg/day) presented higher values of vitamin B_6_ intake than the ANIBES study; Italy, Netherlands, Finland, and France presented lower values of vitamin B_6_ intake than the Spanish elderly from the ANIBES study [14].

In general, vitamin B_6_ food sources include meat and meat products, grains (whole grain corn/maize, brown rice, sorghum, quinoa, wheat germ), pulses, nuts, seeds, potatoes, and some herbs and spices (e.g., garlic, curry, ginger) [14]. In this line, both in European countries [14] and in the ANIBES study, the main source of vitamin B_6_ is meat and meat products. Moreover, while in general, vegetables, cereals/grains, and eggs were included as vitamin B_6_ sources in the ANIBES study, in other European countries grains and milk and dairy products were included.

The biggest strengths of the ANIBES study are the careful design, protocol, and methodology used. Moreover, the random representative sample of the Spanish population aged 9–75 years is also a big strength [26]—the whole population and the plausible energy reporters are taken into account. Some limitations have to be mentioned. The cross-sectional design provides evidence of associations but not causal relationships [24]. Moreover, the low retention data found after the analysis of misreporting is another limitation. Many studies about energy and nutrient intake, apart from national surveys, have an N lower than 500; in this respect, our N of plausible reporters is not negligible. On the other hand, although biotin is involved in the production of energy, we could not include this vitamin in the current analysis. The VD-FEN 2.1 software is based on the food composition tables of Moreiras et al., (2015), which do not include the amount of biotin in food [27]. It has to be mentioned that those participants who were assessed by telephone interview declared a significant lower energy intake (1690 ± 413 kcal/day) than those who recorded with tablet or camera (1824 ± 518 kcal/day, 1834 ± 517 kcal/day, respectively). But, it is important to add that telephone interviews were a minority (6.8%) when compared to tablets (82.4%) and cameras (10.8%), minimizing the bias in the collection of the data.

## 5. Conclusions

The reported intake of thiamin, riboflavin, niacin, and vitamin B_6_ is adequate in the Spanish population according to the ANIBES study. In the whole studied group, 71.2% for thiamin, 72.0% for riboflavin, 99.0% for niacin, and 77.2% for vitamin B_6_ of the population reported intakes that were higher than 80% of the European recommended daily intakes. However, when only the plausible energy reporters are considered, these percentages increased to 93.6%, 91.3% 100%, and 93.4%, respectively. In general, these percentages of subjects meeting the European recommendations decreased with age. Meat and meat products were the main food source intakes for thiamin, niacin, and vitamin B_6_, and milk and dairy products were the main food source intakes for riboflavin.

## Figures and Tables

**Table 1:** Calculated misreporting cut-off at group and individual levels for the Anthropometry, Intake, and Energy Balance in Spain (ANIBES) study.

PAL	Misreporting Cut-off			
	Group Level		Individual Level	
	Lower	Upper	Lower	Upper
Children and adolescents				
1.6	1.55	1.66	0.93	2.76
1.8	1.73	1.86	1.04	3.10
2.0	1.93	2.07	1.16	3.45
Adults and elderly				
1.4	1.38	1.42	0.77	2.53
1.6	1.58	1.62	0.88	2.89
1.8	1.77	2.83	1.00	3.25

**Table 2:** Daily Thiamin, Riboflavin, niacin, and vitamin B_6_ reported intake by sex and age group in the ANIBES Study population.

	Total			Children 9–12 Years			Adolescents 13–17 Years			Adults 18–64 Years			Elderly 65–75 Years		
	*n*	Mean ± SEM	Median (Range)	*n*	Mean ± SEM	Median (Range)	*n*	Mean ± SEM	Median (Range)	*n*	Mean ± SEM	Median (Range)	*n*	Mean ± SEM	Median (Range)
**THIAMIN (mg/day)**															
Total	2009	1.17 ± 0.02	1.07 (0.30–3.44)	213	1.24 ± 0.03	1.20 (0.41–3.32)	211	1.28 ± 0.03	1.24 (0.41–3.01)	1655	1.18 ± 0.02	1.07 (0.30–3.44)	206	1.06 ± 0.03	0.97 (0.40–2.69)
Men	1013	1.27 ± 0.04 *	1.15 (0.33–3.44)	126	1.28 ± 0.04	1.22 (0.41–3.32)	137	1.35 ± 0.04	1.28 (0.41–3.01)	798	1.29 ± 0.05 *	1.16 (0.33–3.44)	99	1.41 ± 0.04	1.00 (0.42–2.69)
Women	996	1.07 ± 0.01	1.00 (0.30–2.70)	87	1.18 ± 0.04	1.15 (0.41–2.70)	74	1.14 ± 0.04	1.17 (0.52–2.31)	857	1.07 ± 0.01	1.01 (0.30–2.68)	107	0.98 ± 0.03	0.89 (0.40–2.17)
**RIBOFLAVIN (mg/day)**															
Total	2009	1.44 ± 0.02	1.34 (0.37–3.54)	213	1.56 ± 0.03	1.54 (0.50–3.41)	211	1.51 ± 0.04	1.43 (0.53–3.82)	1655	1.44 ± 0.02	1.33 (0.40–3.54)	206	1.39 ± 0.04	1.32 (0.37–4.71)
Men	1013	1.53 ± 0.04 *	1.40 (0.42–3.54)	126	1.62 ± 0.04	1.56 (0.50–3.41)	137	1.63 ± 0.05 *	1.51 (0.55–3.82)	798	1.54 ± 0.05 *	1.39 (0.42–3.54)	99	1.47 ± 0.07	1.32 (0.61–4.71)
Women	996	1.35 ± 0.01	1.29 (0.37–3.08)	87	1.49 ± 0.05	1.49 (0.53–2.57)	74	1.29 ± 0.04	1.27 (0.53–2.04)	857	1.36 ± 0.02	1.28 (0.40 + 3.47)	107	1.33 ± 0.05	1.32 (0.37–2.99)
**NIACIN (mg/day)**															
Total	2009	29.1 ± 0.2	27.9 (6.7–109.0)	213	28.5 ± 0.5	27.5 (8.6–58.4)	211	30.2 ± 0.7	30.1 (11.7–73.6)	1655	29.4 ± 0.3	28.1 (6.6–109.0)	206	26.0 ± 0.6	25.0 (9.1–70.3)
Men	1013	31.4 ± 0.3 *	30.2 (9.8–109.0)	126	29.4 ± 0.7	28.9 (8.6–58.4)	137	32.4 ± 0.8 *	32.0 (11.7–73.6)	798	31.9 ± 0.4 *	30.6 (12.1–109.0)	99	28.0 ± 1.0 *	26.2 (9.8–70.3)
Women	996	26.7 ± 0.3	26.1 (6.7–66.1)	87	27.0 ± 0.8	26.1 (13.3–50.6)	74	26.1 ± 0.9	26.2 (12.4–45.3)	857	27.1 ± 0.3	26.4 (6.7–66.1)	107	24.2 ± 07	22.6 (9.1–48.2)
**VITAMIN B_6_ (mg/day)**															
Total	2009	1.54 ± 0.01	1.44 (0.28–9.30)	213	1.51 ± 0.03	1.51 (0.49–4.29)	211	1.54 ± 0.04	1.47 (0.62–3.62)	1655	1.55 ± 0.02	1.44 (0.28–9.30)	206	1.56 ± 0.05	1.39 (0.62–5.29)
Men	1013	1.64 ± 0.02 *	1.51 (0.44–9.30)	126	1.54 ± 0.04	1.54 (0.56–4.29)	137	1.62 ± 0.05 *	1.57 (0.63–3.62)	798	1.65 ± 0.03 *	1.49 (0.44–9.30)	99	1.71 ± 0.08 *	1.51 (0.73–5.29)
Women	996	1.45 ± 0.02	1.37 (0.28–3.94)	87	1.46 ± 0.06	1.46 (0.49–3.28)	74	1.40 ± 0.05	1.31 (0.62–2.86)	857	1.46 ± 0.02	1.39 (0.28–3.94)	107	1.42 ± 0.05	1.29 (0.62–3.61)

Results are expressed as the mean ± standard error of the mean (SEM) and median with range (in brackets); (*) *t*-tests were used to evaluate differences by sex within the whole population and within each age group. Analysis or analyses of variance (ANOVA) tests were used to calculate differences among age groups (mean values within the same row with unlike superscript letters were significantly different). *p* < 0.05 was considered statistically significant.

**Table 3:** Daily thiamin, riboflavin, niacin, and vitamin B_6_ reported intake by plausible reporters, non-plausible reporters, and age group in the ANIBES Study population.

	Total			Children 9–12 Years			Adolescents 13–17 Years			Adults 18–64 Years			Elderly 65–75 Years		
	*n*	Mean ± SEM	Median (Range)	*n*	Mean ± SEM	Median (Range)	*n*	Mean ± SEM	Median (Range)	*n*	Mean ± SEM	Median (Range)	*n*	Mean ± SEM	Median (Range)
**THIAMIN (mg/day)**															
Plausible reporters	543	1.42 ± 0.02	1.32 (0.60–4.88)	120	1.37 ± 0.04	1.31 (0.63–3.32)	76	1.48 ± 0.05	1.43 (060–3.01)	433	1.43 ± 0.03	1.32 (0.65–4.88)	45	1.31 ± 0.05	1.26 (0.74–2.31)
Men	232	1.60 ± 0.03	1.50 (0.63–4.88)	68	1.42 ± 0.05	1.28 (0.63–3.32)	48	1.60 ± 0.06	1.53 (0.93–3.01)	158	1.70 ± 0.05	1.59 (0.83–4.88)	24	1.42 ± 0.09	1.39 (0.90–2.31)
Women	311	1.27 ± 0.02	1.23 (0.60–2.70)	52	1.30 ± 0.05	1.28 (0.79–2.70)	28	1.27 ± 0.07	1.27 (0.60–2.31)	275	1.27 ± 0.02	1.21 (0.65–2.61)	21	1.18 ± 0.05	1.21 (0.74–1.64)
Non-Plausible reporters	1466	1.09 ± 0.02	0.99 (0.30–3.44)	93	1.08 ± 0.04	1.02 (0.41–2.60)	135	1.16 ± 0.03	1.11 (0.41–2.48)	1222	1.09 ± 0.03	0.99 (0.33–3.44)	161	0.99 ± 0.03	0.89 (0.40–2.69)
Men	781	1.18 ± 0.04	1.07 (0.33–3.44)	58	1.12 ± 0.06	1.01 (0.41–2.60)	89	1.22 ± 0.05	1.15 (0.41–2.48)	640	1.19 ± 0.05	1.08 (0.3–3.4)	75	1.05 ± 0.05	0.97 (0.42–2.69)
Women	685	0.98 ± 0.01	0.92 (0.03–2.68)	35	1.01 ± 0.06	1.04 (0.41–1.83)	46	1.06 ± 0.04	1.07 (0.52–1.68)	582	0.98 ± 0.01	0.92 (0.30–2.68)	86	0.93 ± 0.04	0.85 (0.40–2.17)
**RIBOFLAVIN (mg/day)**															
Plausible reporters	543	1.74 ± 0.02	1.65 (0.69–6.13)	120	1.72 ± 0.04	1.67 (0.96–3.41)	76	1.79 ± 0.06	1.77 (0.78–3.46)	433	1.73 ± 0.03	1.63 (0.9–6.13)	45	1.77 ± 0.10	1.68 (0.85–4.18)
Men	232	1.93 ± 0.03	1.84 (0.92–6.13)	68	1.77 ± 0.05	1.73 (1.04–3.41)	48	1.95 ± 0.08	1.92 (1.09–3.46)	158	1.98 ± 0.05	1.88 (0.93–6.13)	24	1.96 ± 0.14	1.72 (0.92–4.18)
Women	311	1.59 ± 0.02	1.54 (0.69–3.08)	52	1.65 ± 0.06	1.58 (0.96–2.57)	28	1.52 ± 0.06	1.51 (0.78–2.04)	275	1.59 ± 0.03	1.52 (0.69–3.08)	21	1.56 ± 0.11	1.56 (0.85–2.99)
Non-Plausible reporters	1466	1.34 ± 0.02	1.25 (0.37–3.53)	93	1.36 ± 0.04	1.35 (0.50–3.04)	135	1.35 ± 0.04	1.34 (0.53–3.82)	1222	1.34 ± 0.03	1.24 (0.40–3.54)	161	1.29 ± 0.04	1.22 (0.37–4.71)
Men	781	1.42 ± 0.04	1.30 (0.42–3.53)	58	1.43 ± 0.06	1.35 (0.50–3.04)	89	1.45 ± 0.06	1.38 (0.55–3.82)	640	1.43 ± 0.06	1.30 (0.42–3.54)	75	1.31 ± 0.07	1.20 (0.61–4.71)
Women	685	1.25 ±0.02	1.19 (0.37–2.47)	35	1.25 ± 0.07	1.32 (0.53–1.83)	46	1.15 ± 0.05	1.09 (0.53–1.84)	582	1.25 ± 0.02	1.18 (0.40–3.47)	86	1.27 ± 0.05	1.25 (0.37–2.61)
**NIACIN (mg/day)**															
Plausible reporters	543	34.1 ± 0.4	33.0 (13.3–105.9)	120	31.2 ± 0.6	31.1 (18.8–58.4)	76	34.0 ± 1.1	33.1 (13.9–68.3)	433	35.1 ± 0.5	33.8 (13.3–105.9)	45	32.6 ± 1.5	32.9 (16.0–70.3)
Men	232	38.1 ± 0.6	36.1 (16.0–105.8)	68	32.4 ± 0.8	31.6 (18.8–58.4)	48	37.3 ± 1.2	35.7 (17.7–68.3)	158	41.1 ± 1.0	39.0 (19.8–105.9)	24	35.7 ± 2.3	34.1 (16.0–70.3)
Women	311	30.9 ± 0.4	30.7 (13.3–66.1)	52	29.7 ± 1.0	28.4 (19.5–50.6)	28	28.2 ± 1.5	28.2 (13.9–45.3)	275	31.6 ± 0.5	30.8 (13.3–66.1)	21	29.0 ± 1.5	29.8 (16.6–39.9)
Non-Plausible reporters	1466	27.0 ± 0.2	25.8 (6.7–109.0)	93	25.0 ± 0.8	24.3 (8.6–46.1)	135	28.1 ± 0.8	27.6 (11.7–73.6)	1222	27.4 ± 0.3	26.1 (6.7–109.0)	161	24.2 ± 0.6	22.8 (9.1–61.8)
Men	781	29.1 ± 0.3	27.6 (8.6–1090.)	58	26 ± 1	26 (9–46)	89	29.7 ± 1.0	28.5 (11.7–73.6)	640	29.6 ± 0.4	28.1 (12.1–109.0)	75	25.5 ± 0.9	24.9 (9.8–61.8)
Women	685	24.7 ± 0.3	24.1 (6.7–53.2)	35			46	24.9 ± 1.1	24.5 (12.4–43.5)	582	25.0 ± 0.3	24.6 (6.7–53.2)	86	23.1 ± 0.7	21.9 (9.1–48.2)
**VITAMIN B_6_ (mg/day)**															
Plausible reporters	543	1.83 ± 0.03	1.72 (0.57–7.71)	120	1.66 ± 0.04	1.63 (0.83–4.29)	76	1.80 ± 0.06	1.81 (0.62–3.62)	433	1.85 ± 0.03	1.73 (0.57–7.71)	45	2.06 ± 0.12	1.89 (0.99–5.29)
Men	232	2.05 ± 0.04	1.90 (0.85–7.71)	68	1.71 ± 0.06	1.68 (0.87–4.29)	48	1.92 ± 0.07	1.86 (1.05–3.62)	158	2.20 ± 0.07	2.06 (0.85–7.71)	24	2.35 ± 0.19	2.11 (1.12–5.29)
Women	311	1.7 ± 0.0	1.65 (0.57–3.94)	52	1.58 ± 0.06	1.53 (0.83–3.16)	28	1.60 ± 0.10	1.49 (0.62–2.86)	275	1.66 ± 0.03	1.60 (0.57–3.94)	21	1.74 ± 0.10	1.71 (0.99–3.01)
Non-Plausible reporters	1466	1.43 ± 0.01	1.33 (0.28–9.30)	93	1.32 ± 0.05	1.30 (0.49–3.28)	135	1.39 ± 0.04	1.34 (0.63–3.16)	1222	1.44 ± 0.02	1.33 (0.28–9.30)	161	1.42 ± 0.04	1.31 (0.62–3.72)
Men	781	1.49 ± 0.02	1.39 (0.44–9.30)	58	1.34 ± 0.06	1.37 (0.56–2.50)	89	1.46 ± 0.06	1.39 (0.63–3.16)	640	1.51 ± 0.03	1.39 (0.44–9.30)	75	1.50 ± 0.06	1.39 (0.73–3.72)
Women	685	1.35 ± 0.02	1.26 (0.28–3.61)	35	1.28 ± 0.09	1.25 (0.49–3.28)	46	1.28 ± 0.05	1.2 (0.70–2.21)	582	1.36 ± 0.02	1.28 (0.28–3.17)	86	1.34 ± 0.05	1.21 (0.62–3.61)

Results are expressed as the mean standard error of the mean and median with range (in brackets). There were significant differences between plausible and non-plausible reporters for the whole population and within sexes into each age group (*p* < 0.05), except for vitamin B_6_ in the children, adults and elderly.

**Table 4:** Percentage of the population with adequate intake of thiamin, riboflavin, niacin, and vitamin B_6_, for the whole population and for the plausible energy reporters by age groups.

	Total	Children 9–12 Years	Adolescents 13–17 Years	Adults 18–64 Years	Elderly 65–75 Years
	*EFSA*	*EFSA*	*EFSA*	*EFSA*	*EFSA*
**THIAMIN (%)**					
Whole population	71.2	91.5	77.7	70.8	61.2
Men	75.9	92.9	81.8	74.9	70.7
Women	66.5	89.7	70.3	67.0	52.3
Plausible reporters	93.6	99.2	90.8	91.9	93.3
Men	98.3	98.5	95.8	97.5	100.0
Women	90.0	100.0	82.1	88.7	85.7
**RIBOFLAVIN (%)**					
Whole population	72.0	92.5	83.4	70.2	66.5
Men	75.4	95.2	86.9	74.3	67.7
Women	68.5	88.5	77.0	66.4	65.4
Plausible reporters	91.3	100.0	98.7	89.6	84.4
Men	97.8	100.0	100.0	97.5	95.8
Women	68.5	100.0	96.4	85.1	71.4
**NIACIN (%)**					
Whole population	99.0	100.0	99.5	98.9	97.6
Men	99.6	100.0	100.0	99.7	98.0
Women	98.3	100.0	98.6	98.1	97.2
Plausible reporters	100.0	100.0	100.0	100.0	100.0
Men	100.0	100.0	100.0	100.0	100.0
Women	100.0	100.0	100.0	100.0	100.0
**VITAMIN B_6_ (%)**					
Whole population	77.2	89.7	83.4	76.0	78.6
Men	79.9	92.1	87.6	78.2	86.9
Women	74.5	86.2	75.7	74.0	71.0
Plausible reporters	93.4	97.5	93.4	91.9	97.8
Men	99.1	98.5	100.0	99.4	100.0
Women	89.1	96.2	82.1	87.6	95.2

Results are expressed in percentage. Recommended daily intakes for Europe for thiamin [4], riboflavin [8], niacin [12], and vitamin B_6_ [15]. Adequacy intake was calculated comparing with 80% of the EFSA PRI or AI.

## Supplementary Materials

The following are available online at http://www.mdpi.com/2072-6643/10/7/846/s1. Figure S1. Contribution of food sources to the daily thiamin, riboflavin, niacin vitamins B_6_ intake in the ANIBES children. Figure S2. Contribution of food sources to the daily thiamin, riboflavin, niacin vitamins B_6_ intake in the ANIBES adolescents. Figure S3. Contribution of food sources to the daily thiamin, riboflavin, niacin vitamins B_6_ intake in the ANIBES adults. Figure S4. Contribution of food sources to the daily thiamin, riboflavin, niacin vitamins B_6_ intake in the ANIBES elderly.
