# Supplementary material for: Dietary Intake and Food Sources of Niacin, Riboflavin, Thiamin and Vitamin B6 in a Representative Sample of the Spanish Population. The ANIBES Study
Source: Nutrients. 2018 Jun 29;10(7):846. doi: 10.3390/nu10070846 (PMC6073544; doi:10.3390/nu10070846)

Figure S1. Contribution of food sources to the daily thiamin, riboflavin, niacin vitamins B6 intake in the ANIBES children.

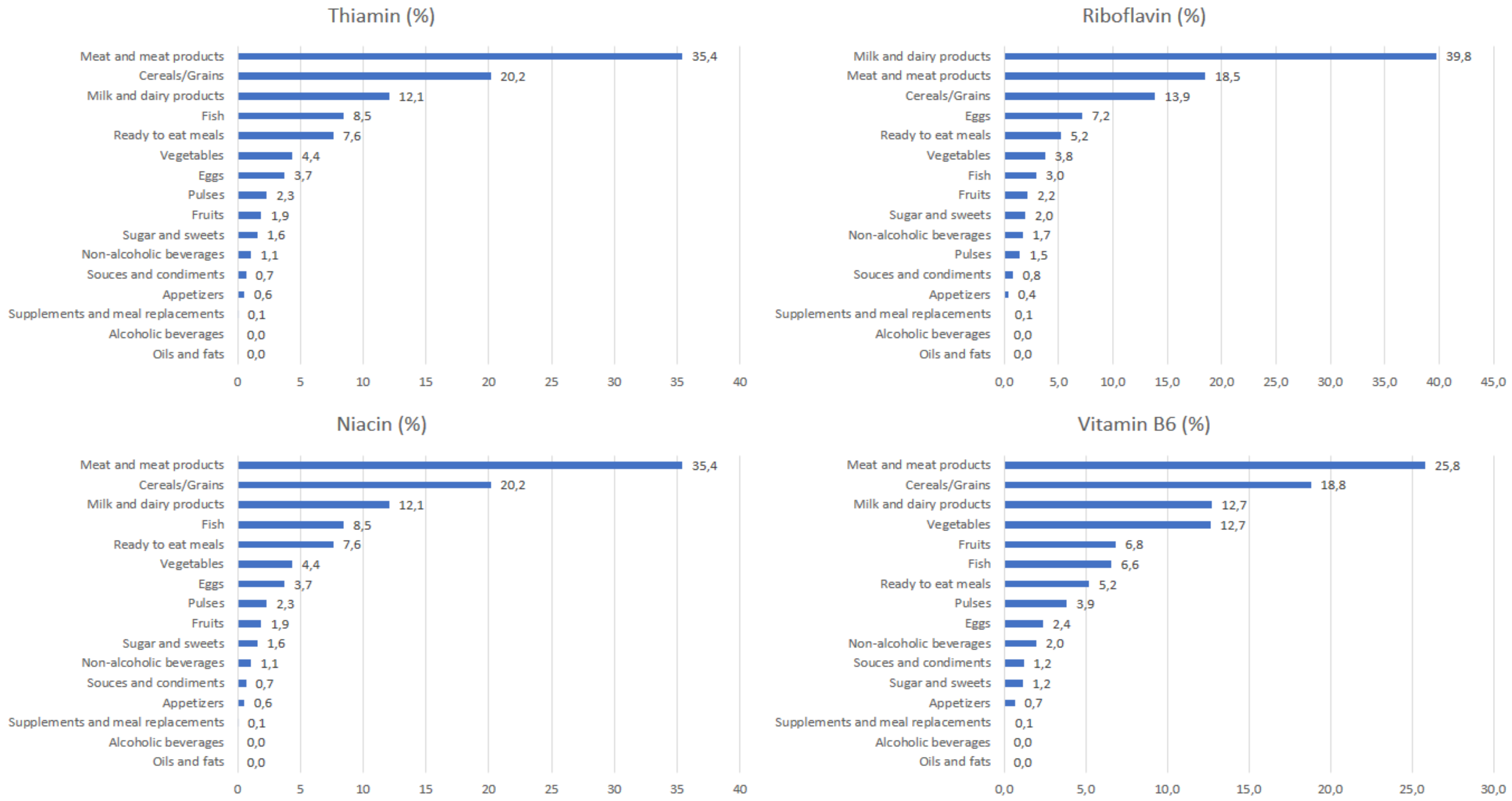

Figure S2. Contribution of food sources to the daily thiamin, riboflavin, niacin vitamins B6 intake in the ANIBES adolescents.

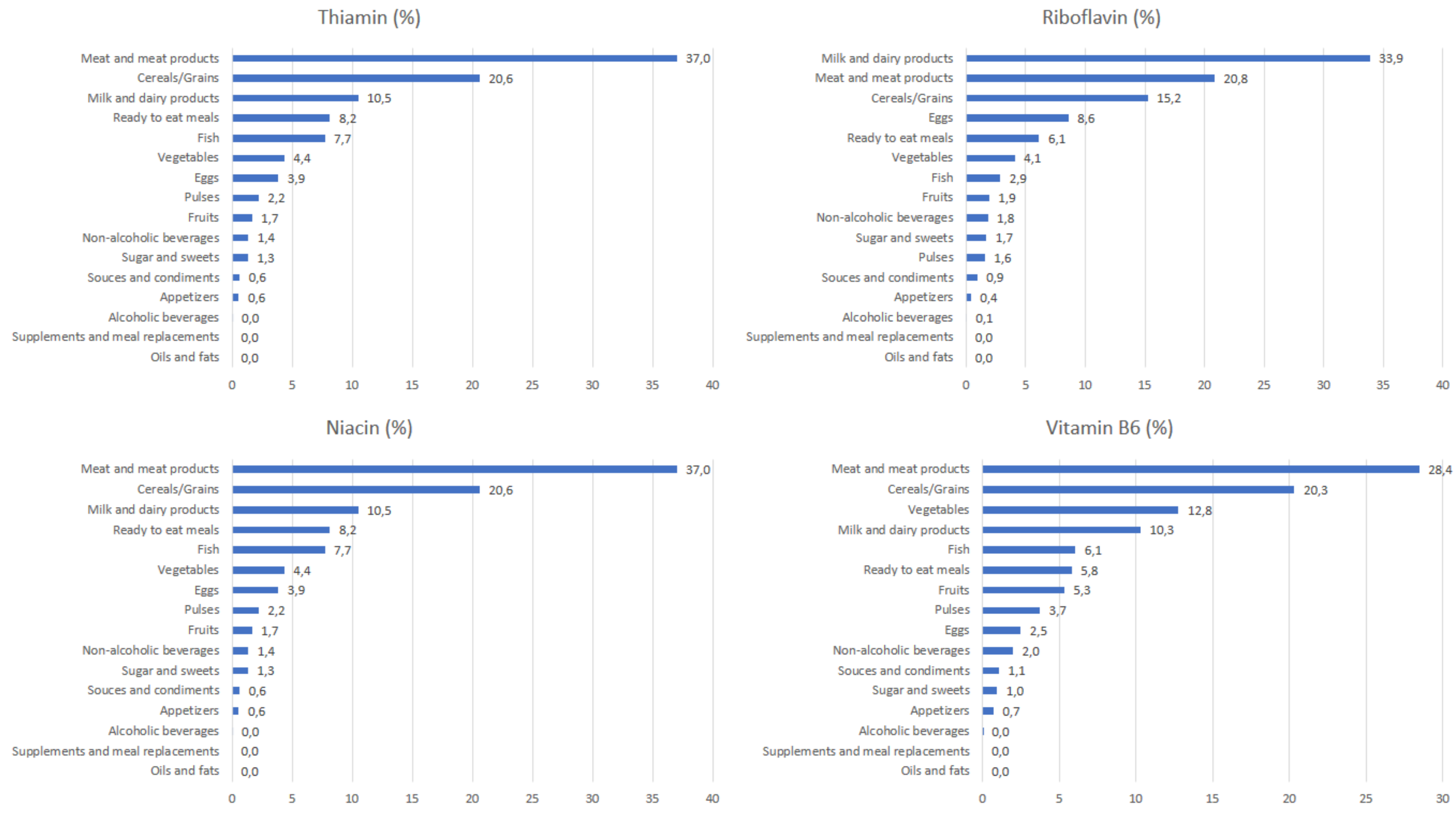

Figure S3. Contribution of food sources to the daily thiamin, riboflavin, niacin vitamins B6 intake in the ANIBES adults.

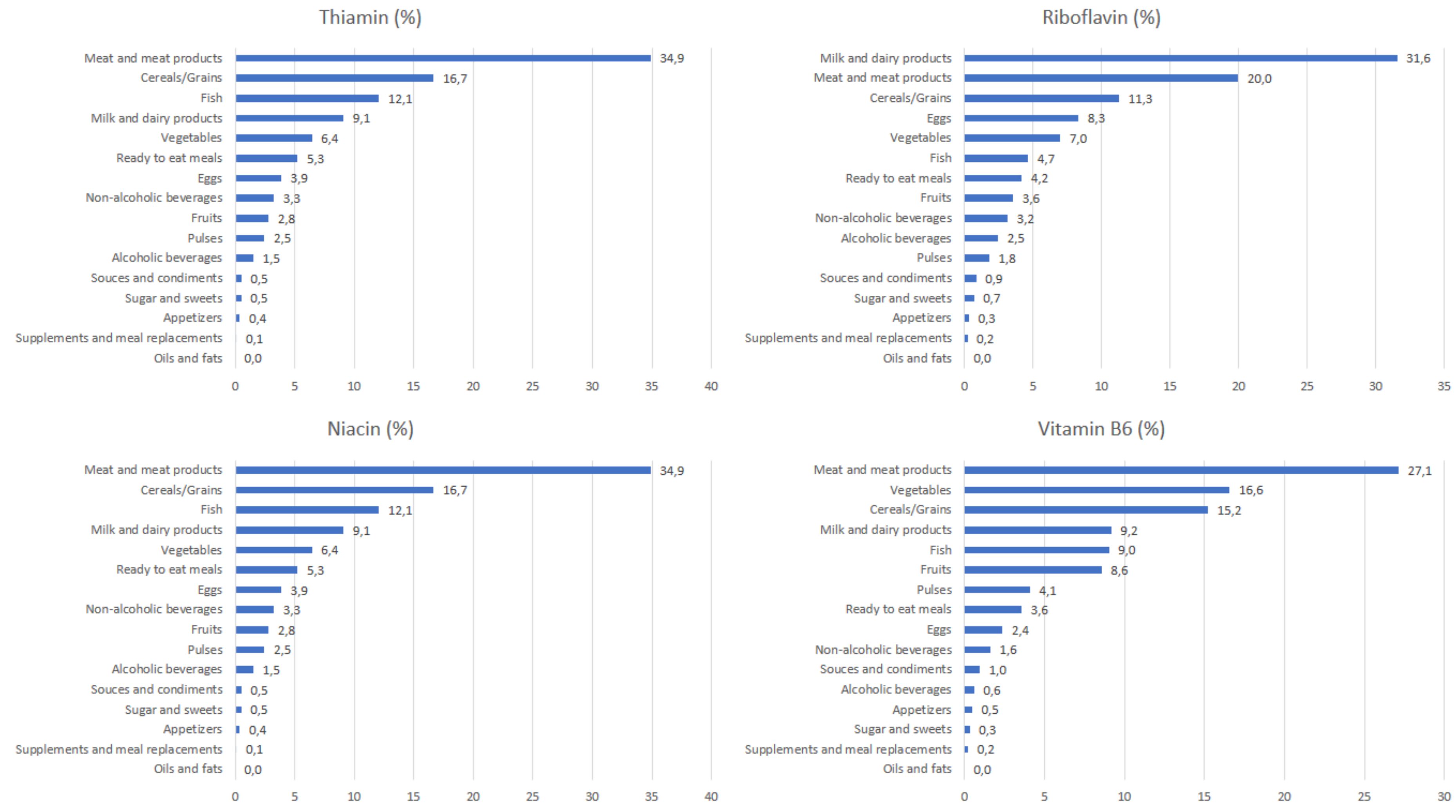

Figure S4. Contribution of food sources to the daily thiamin, riboflavin, niacin vitamins B6 intake in the ANIBES elderly.

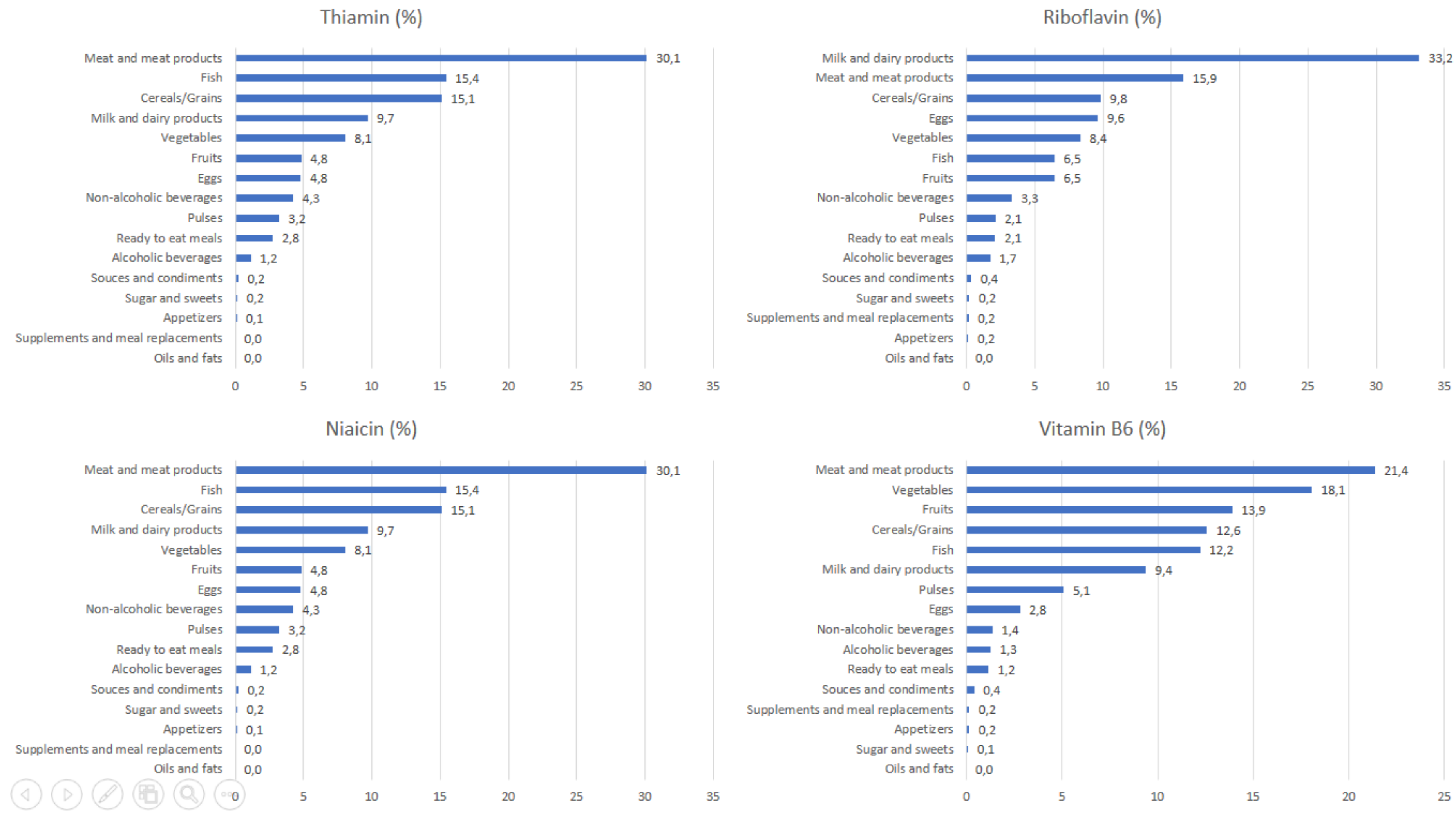

Supplement: Supplementary file 1 [file nutrients-10-00846-s001.pdf]
